# A FACS-Free Purification Method to Study Estrogen Signaling, Organoid Formation, and Metabolic Reprogramming in Mammary Epithelial Cells

**DOI:** 10.3389/fendo.2021.672466

**Published:** 2021-08-12

**Authors:** Aurélie Lacouture, Cynthia Jobin, Cindy Weidmann, Line Berthiaume, Dominic Bastien, Isabelle Laverdière, Martin Pelletier, Étienne Audet-Walsh

**Affiliations:** ^1^Endocrinology - Nephrology Research Axis, CHU de Québec - Université Laval Research Center, Québec City, QC, Canada; ^2^Department of Molecular Medicine, Faculty of Medicine, Université Laval, Québec City, QC, Canada; ^3^Centre de recherche sur le cancer de l’Université Laval, Québec City, QC, Canada; ^4^Faculty of Pharmacy, University Laval, Quebec City, QC, Canada; ^5^Oncology Axis, Centre de recherche du CHU de Québec - Université Laval, Quebec City, QC, Canada; ^6^Department of Pharmacy, CHU de Québec-Université Laval, Quebec City, QC, Canada; ^7^Infectious and Immune Disease Axis, CHU de Québec-Université Laval Research Center, Québec, QC, Canada; ^8^ARThrite Research Center, Laval University, Québec, QC, Canada; ^9^Department of Microbiology-Infectious Diseases and Immunology, Faculty of Medicine, Laval University, Québec, QC, Canada

**Keywords:** nuclear receptor, estrogen receptor, steroid, breast cancer, organoids, lactation, metabolomics, breast feeding

## Abstract

Few *in vitro* models are used to study mammary epithelial cells (MECs), and most of these do not express the estrogen receptor α (ERα). Primary MECs can be used to overcome this issue, but methods to purify these cells generally require flow cytometry and fluorescence-activated cell sorting (FACS), which require specialized instruments and expertise. Herein, we present in detail a FACS-free protocol for purification and primary culture of mouse MECs. These MECs remain differentiated for up to six days with >85% luminal epithelial cells in two-dimensional culture. When seeded in Matrigel, they form organoids that recapitulate the mammary gland’s morphology *in vivo* by developing lumens, contractile cells, and lobular structures. MECs express a functional ERα signaling pathway in both two- and three-dimensional cell culture, as shown at the mRNA and protein levels and by the phenotypic characterization. Extracellular metabolic flux analysis showed that estrogens induce a metabolic switch favoring aerobic glycolysis over mitochondrial respiration in MECs grown in two-dimensions, a phenomenon known as the Warburg effect. We also performed mass spectrometry (MS)-based metabolomics in organoids. Estrogens altered the levels of metabolites from various pathways, including aerobic glycolysis, citric acid cycle, urea cycle, and amino acid metabolism, demonstrating that ERα reprograms cell metabolism in mammary organoids. Overall, we have optimized mouse MEC isolation and purification for two- and three-dimensional cultures. This model represents a valuable tool to study how estrogens modulate mammary gland biology, and particularly how these hormones reprogram metabolism during lactation and breast carcinogenesis.

## Introduction

The mammary gland is known to be highly sensitive to sex-steroid hormones such as estradiol (E_2_), the most potent endogenous estrogen. Indeed, E_2_ is critical for development of the mammary gland and its evolution through the estrous and reproduction cycles (1, 2). The two estrogen receptors, ERα and ERβ, are transcription factor members of the nuclear receptor family expressed in most tissues in both males and females, including luminal epithelial cells of the mammary gland (3). Interestingly, sex-steroid hormone receptors are increasingly recognized as modulators of cell metabolism (4), even though it is still unknown how ERs reprogram cell metabolism in the mammary gland.

Following pregnancy, lactation in mammals represents a major energy investment. The mammary gland must sustain milk production and is thus a highly active metabolic tissue (1). Mammary luminal epithelial cells produce and secrete milk, which consists of water, proteins, lipids, and carbohydrates (lactose) that are secreted mainly as secretory vesicles. These epithelial cells uptake nutrients from the blood and metabolize them in a specific manner to produce the milk constituents. All the major milk proteins, such as caseins, are synthesized by mammary epithelial cells (MECs) from amino acids, with some exceptions such as serum albumin and immunoglobulins. Consequently, during lactation, the mammary gland exhibits high amino acid uptake and metabolism linked to protein synthesis (1). Branched-chain amino acids are catabolized extensively in this tissue through various cytoplasmic and mitochondrial metabolic pathways (1). Similarly, blood glucose is converted into lactose, and lipids are synthesized in these cells and secreted by secretory vesicles (5). Thus, a specific metabolic program must be regulated in the mammary gland to sustain its specific functions during lactation.

The different regulatory pathways that govern metabolism are mostly unknown at the cellular level. Historically, ERα was mainly associated with the regulation of biological pathways linked to mammary gland development and has not been considered a major metabolic modulator at the cellular level. This contrasts with the well-known relationship between estrogen exposure and whole-body metabolic homeostasis (6, 7). ERs activation has been shown to modulate metabolism in breast cancer cells, notably by differentially altering mitochondrial properties such as respiration, fusion, and fission (8–12). The sparse data available to date mostly come from breast cancer models and not from normal mammary epithelial cells (6, 7). Because metabolic reprogramming is a hallmark of cancer, data from cancer models are most probably not transposable to normal MEC, further highlighting the need to address this question.

Immortalized cell lines from the mammary gland have little or no ERα expression, including the most widely used models, the MCF10A and hTERT cell lines (13, 14). The use of primary mammary epithelial cells is an attractive alternative and is expected to better recapitulate physiological conditions. Still, most published methods to purify these cells often require flow cytometry and fluorescence-activated cell sorting (FACS), which are time-consuming, require specialized instruments, and necessitate specific expertise (15–17). In the current study, we aimed to optimize a FACS-free protocol to purify mammary epithelial cells for two- and three-dimensional culture studies to study ERα metabolic functions. Herein, this protocol for both purification of MECs and their primary culture is described in depth. In addition, we demonstrate that MECs cultured in two- and three-dimensions retain a functional ERα signaling pathway and that they can be used for state-of-the-art metabolomic studies.

## Material and Methods

### Primary Mammary Epithelial Cell Purification

Mice were bred, housed, and handled at the animal facility of the Centre de recherche du CHU de Québec – Université Laval. C57BL/6 mice were kept in a 12h light:12h dark cycle at 22°C and all protocols were performed according to the Université Laval Research and Ethic Animal Committee’s guidelines and regulations.

All the volumes described below are for the purification of mammary epithelial cells from three female mice with an average age of 20 weeks. After sacrifice, the two thoracic and two inguinal mammary glands were collected and conserved on ice in 1X complete HBSS solution (HBSS + 2% FBS + 10 mM HEPES + 100 U/ml penicillin and 100 µg/ml streptomycin (Wisent)) before being cut in small pieces with scissors under a biological hood. The tissues were then transferred to a tube containing a 1X solution of Gentle Collagenase/Hyaluronidase (StemCell) with 1X complete EpiCult-B mouse medium + 5% FBS (EpiCult basal medium (StemCell) + EpiCult proliferation supplement (StemCell) + 10 ng/ml Recombinant human EGF (StemCell) + 10 ng/ml Recombinant human bFGF (StemCell) + 4 µg/ml heparin (Sigma) + 100 U/ml penicillin and 100 µg/ml streptomycin). After overnight incubation at 37°C without shaking, the solution was centrifuged at 350 g for 7 min. The supernatant was discarded by pipetting and the pellet was washed with 1X HBSS complete solution to remove residual fat. The solution was centrifuged at 350 g for 5 min, and the supernatant was discarded by pipetting. The pellet was resuspended with 1 ml of warm 0.25% trypsin-EDTA (Wisent). After 3 min of gentle pipetting, 4 ml of 0.25% trypsin-EDTA was added to obtain a total of 5 ml and then kept on ice for 1 h. After adding 10 ml of 1X complete HBSS solution, the tube was inverted 2-3 times to gently mix the suspension and then centrifuged at 350 g for 5 min. The supernatant was removed by pipetting, and 2 ml of warm dispase (5U/ml – StemCell) and 0.1-1 mg/ml de DNase I (Roche) were added. Clumps were dissociated by pipetting for 1-3 min, then 10 ml of cold 1X complete HBSS solution was added. The tube was gently inverted 2-3 times, and the suspension was filtered through a 40 µm Cell Strainer (Falcon). The filtered suspension was centrifuged at 350 g for 5 min, and cells were counted. The purification process was performed using the EasySep Mouse Epithelial Cell Enrichment kit II (StemCell) according to the manufacturer’s protocol, after which, the cells were counted and plated for 1 h for differential plating at 37°C. After this incubation, the media, which contains epithelial cells that require more time to adhere, was removed and transferred to a new plate. The new plate contained the purified mouse mammary epithelial cells.

### Primary Cell Culture Conditions

After purification and differential plating, cells were plated in complete EpiCult-B mouse medium (StemCell) + 5% FBS in a 37°C incubator with 5% CO_2_. After 24 h, the medium was replaced by serum-free complete EpiCult-B mouse medium and changed every three days. For three-dimensional culture, the cell suspension obtained after differential plating was centrifuged at 350 g for 5 min. Cells were resuspended in complete EpiCult-B mouse medium with 5% FBS and 75% growth factor reduced Matrigel (Corning) to obtain a droplet. Each droplet contained 30 000 cells in 40 µl of cell suspension + Matrigel and was seeded into a warm 24-well-plate using cold tips. The plate was then turned upside down and incubated for 15 min at 37°C. Finally, the plate was turned upright and 500 µl of warm complete EpiCult-B mouse medium + 5% FBS was added to each well. After 24 h the medium was replaced by a serum-free medium that was changed every three days.

### Cell Labeling and Flow Cytometry Analysis

Before and after purification, cells were resuspended in PBS with 5% FBS and stained using CD24-FITC (1:300, M1/69, BioLegend) and CD49f-PE (1:30, GoH3, BD Biosciences). Antibodies were incubated for 30 min at 4°C and then cells were washed twice in PBS + 5% FBS. Data were acquired using a BD FACSCelesta™ Flow Cytometer and BD FACSDiva™ software version 8.0.1.1 (Becton, Dickinson and Company). Data analyses were performed using FlowJo™ version 10.7.1 (Becton, Dickinson and Company, 2019). Ten thousand events were acquired for each sample. Cells were first gated by their forward and side scatter, representing cell size and granularity. Luminal and basal epithelial cells were distinguished respectively using CD24 and CD49f markers.

### Immunofluorescence + Microscopy Analysis

The protocol for immunofluorescence was used as previously described (18). Briefly, cells were cultured in Nunc™ Lab-Tek™ II Chamber slide™ system (ThermoFisher Scientific). After three or six days in culture, the cells were fixed with 4% paraformaldehyde for 15 min. Cells were permeabilized for 5 min with 0.5% Triton-PBS. The primary antibodies for CK8/18 (1:1000, MA5-32118, Invitrogen) or vimentin (1:200, 5741T, Cell Signaling) were incubated overnight in 1% FBS-PBS at 4°C. After washing, secondary antibodies (α-rabbit 488 and 555, 1:2000, A-11008 and A-21428, Invitrogen) were incubated for 1 h at room temperature. Finally, Fluoromount + DAPI (ThermoFisher Scientific) were added. For the immunofluorescence and microscopy analyses, images were taken with the EVOS™ M5000 Imaging System (ThermoFisher Scientific) and analyzed using the ImageJ software. Student’s T-test was used to evaluate statistical significance.

### RNA Extraction and Quantitative Reverse Transcription PCR

For epithelial cells grown in two-dimensions, medium was changed after 2 days in culture and cells were treated with 10 nM E_2_ or with 96% EtOH as a control, as previously performed (19). After 24 h of treatment, they were harvested for RNA purification using the RNeasy purification kit (QIAGEN) following the manufacturer’s instructions. For organoids, media was changed after 11 days in culture and cells were treated with or without 10 nM E_2_. The next day, they were collected in cold PBS and centrifuged at 350 g for 5 min to remove the Matrigel, and RNA was purified as for cells grown in two-dimensions. After purification, RNA was used to synthesize cDNA with the LunaScript RT SuperMix Kit (New England Biolabs). Quantitative RT-PCR (qRT-PCR) was then performed on cDNA samples with the Luna Universal qPCR Master Mix (New England Biolabs) for specific quantification of genes, with duplicate technical replicates performed for every sample. To obtain relative gene expression, normalization was performed using the expression of three housekeeping genes, *Pum1*, *Tbp*, and *Actn*. The primers used can be found in **Supplementary Table S1**. Results are shown as the average of three independent experiments with at least three biological replicates per condition. Student’s T-test was used to evaluate statistical significance.

### Western Blot

For protein analyses, buffer K was used to obtain whole-cell lysates (WCLs), as described previously (19). WCLs were analyzed by western blots using primary antibodies: CK8/18 (1:1 000, SU0338, Invitrogen), Vimentin (1:1 000, D21H3, Cell Signaling), ERα (1:1 000, E115, Abcam), Tubulin (1:5 000, 11H10, Cell Signaling Technology), and S6 (1:1 000, C-8, Santa Cruz Biotechnology).

### Extracellular Flux Analyses

Purified mammary gland epithelial cells (40 000 per well) were plated in a Seahorse XF96e microplate with 200 µl of complete EpiCult-B mouse medium + 5% FBS. After 24 h, the medium was replaced by a complete EpiCult-B mouse medium and cells were treated with vehicle (EtOH 96%) or 10 nM E_2_. After 48 h of treatment, cells were rinsed with Seahorse XF assay medium (RPMI with no phenol red) and a final volume of 175 μl of Seahorse XF assay medium was added to each well. After a 1 h equilibration at 37°C in a CO_2_-free incubator, the XF96e microplate was inserted into the XF96e instrument for measurements of oxygen consumption and extracellular acidification rates, as previously described (20–22). Student’s T-test was used to evaluate statistical significance, with *p*<0.05 considered as significant.

### Gaz Chromatography – Mass Spectrometry

Organoids were washed in ice-cold saline and harvested on dry ice with dry ice-cold 80% MeOH. The cells were then lysed by sonication and centrifuged at 20 000 *g* for 10 min (23). In parallel, cell culture media was also harvested for analysis and 400 μl of dry ice-cold 80% MeOH was mixed with 200 μl of culture media before centrifugation. After centrifugation of both media and organoids, the supernatant was transferred into a clean tube containing the internal standard Myristic acid-d27 (CDN isotopes, Canada), to which 700 μl ACN was added. Samples were then vortexed, centrifuged, and the supernatant was recovered to be dried using nitrogen gas. Subsequently, a two-step derivatization was performed according to the method described by Fiehn et al. (24) for methoxiamination, and the modified method from Patel et al. (25) for silylation with MTBSTFA/TBDMCS (Sigma Aldrich, MO, USA; TCI America, Cambridge, MA, USA). Samples were then used for gas chromatography - mass spectrometry (GC-MS) analysis using an Agilent 8890 GC equipped with DB5-MS+DG capillary column connected to an Agilent 5977B MS operating under electron impact (EI) ionization at 70 eV (Agilent Technologies, Santa Clara, CA, USA). One µl of sample was injected in split mode at 250°C, using helium as the carrier gas at a flow rate of 1 ml/min. The GC oven temperature was held at 50°C for 2 min, then was raised from 50 to 150°C at a rate of 20°C/min for 5 min, and from 150 to 300°C at a rate of 10°C/min; the column temperature was then kept constant at 300°C for another 10 min. The MS source and quadrupole were held at 230°C and 150°C, respectively, and the detector was operated in scanning for mass range 50-600 Da at a signal rate 5,1 scans/sec. Agilent MassHunter Workstation Software was used for analysis (Agilent Technologies, CA, USA). Metabolites were found by deconvolution and identified according to spectral match in the NIST/EPA/NIH Mass Spectral Library (NIST 2017, Gaithersburg, MD, USA). Standards for TCA cycle intermediates were used in parallel to perform absolute quantification; other metabolites are shown in relative quantification normalized with myristic acid-d27. Student’s T-test was used to evaluate statistical significance between conditions.

## Results

### Isolation and Purification of Mouse Mammary Epithelial Cells

**Figure 1:**
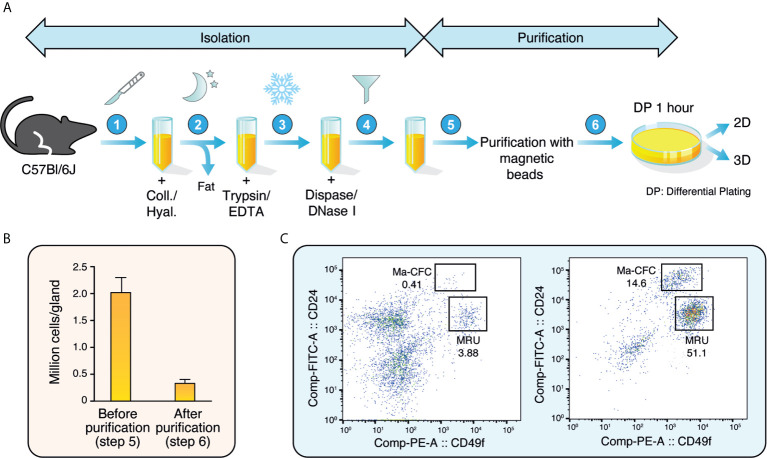
Isolation and purification of mouse mammary epithelial cells. **(A)** Schematic representation of the isolation and purification protocols to obtain mouse mammary epithelial cells. **(B)** Number of mammary epithelial cells obtained before and after isolation and purification (between steps 5 and 6 in A). Results are shown as mean ± SEM of 11 independent experiments. **(C)** Distribution of mammary epithelial cells before (left) and after (right) purification according to their CD24 and CD49f expression. The mammary colony-forming cell (Ma-CFC) fraction was defined as the CD24^High^; CD49f^Low^ and the mammary repopulating units (MRU) fraction as the CD24^Low^; CD49f^High^. Numbers are percentages. One representative experiment out of three independent experiments is shown.

In the mammary gland, basal epithelial cells form an outer layer surrounding an inner layer of luminal epithelial cells that face the lumen where milk is secreted. These luminal cells comprise both ERα-positive (ERα+) and ERα-negative (ERα-) cells that will form lobular-alveolar structures and will be responsible for secreting milk following pregnancy. During lactation, the epithelial cell compartment expands and forms alveoli, leading to milk production (26). The protocol described herein was designed to isolate these epithelial cells by combining an isolation protocol based on enzymatic digestion followed by cell-specific purification using magnetic beads coupled with specific antibodies and differential plating (**Figure 1A**).

After mechanical dissociation, a slow, gentle dissociation with a mix of collagenase and hyaluronidase was used to dissociate the fat pad and the epithelial branching structures. Secondly, cell-cell interactions were inactivated with trypsin and dispase. DNase I was used to eliminate DNA released by cell death. The cells were then filtered to obtain a single-cell suspension.

The single-cell suspension was incubated in a mix of non-epithelial-specific antibodies coupled to magnetic beads, leading to the capture of non-epithelial cells on the magnetic beads. The suspension is thus enriched in epithelial cells, with a four-fold decrease in total cell numbers after this purification step (**Figure 1B**). To confirm that the epithelial cell population was enriched, the single-cell suspension was examined by flow cytometry before and after purification. Antibodies targeting CD24 and CD49f, markers of luminal and basal epithelial cells, respectively, were used. As shown in **Figure 1C**, less than 5% of all cells were strongly positive for either marker before purification. After purification, we obtained a >10-fold enrichment of mammary epithelial cells. The two well-known epithelial cell populations were observed: the mammary colony-forming cells (Ma-CFC) or luminal cells, which are CD24^High^; CD49^Low^, and the mammary repopulating units (MRU) or basal cells which are CD24^Low^; CD49f^High^ (27). Overall, these results showed that our isolation protocol significantly enriches mammary epithelial cells.

### Primary Culture of Mouse Mammary Epithelial Cells in Two-Dimensions

**Figure 2:**
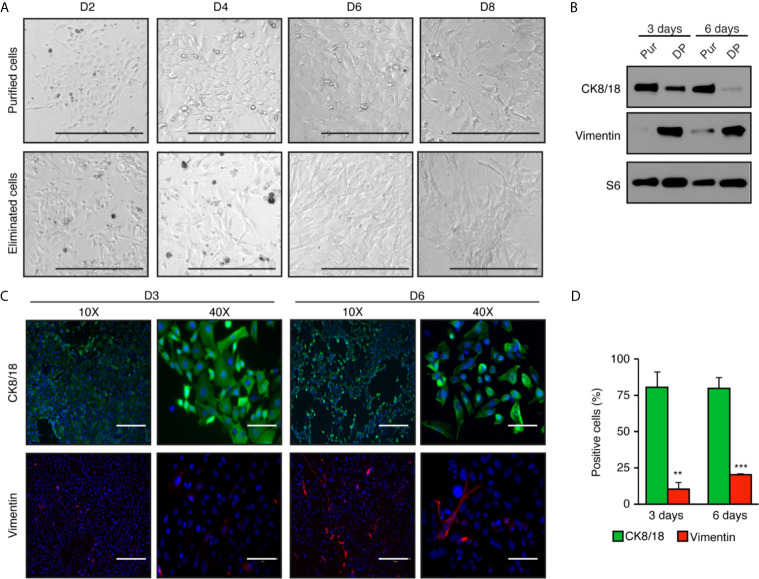
High enrichment of mammary epithelial cell for primary culture in two-dimensions. **(A)** Brightfield images of mammary epithelial cells obtained after purification compared with the cells eliminated during the purification process. Scale bars = 300 μm. **(B)** Western blot analysis of purified cells normally discarded with differential plating (DP) compared to purified epithelial cells (Pur). Protein expression of the cytokeratin 8 and 18 (CK8/18), a marker of epithelial cells, and vimentin, a marker of fibroblasts, after three and six days in two-dimensional culture. S6 was used as the loading control. **(C)** Immunofluorescence showing the expression of CK8/18 (green) and vimentin (red) at three or six days in two-dimensional culture. Nuclei were stained with DAPI (blue). Scale bars = 300 μm and 75 μm. **(D)** Ratios of positive cells for CK8/18 or vimentin per the total number of cells (counts of nuclei) in percentage. Data are shown as mean ± SEM of one representative experiment (n = 6 images per condition). **p < 0.01; ***p < 0.001.

After purification of primary mouse mammary epithelial cells, we proceeded to differential plating (**Figure 1A**). Stromal cells like fibroblasts need <30 min to attach while epithelial cells need >60 min. Consequently, after purification cells were plated for an hour, and then the supernatant with the non-attached (epithelial) cells was collected. The supernatant was then transferred to a new plate, where epithelial cells were allowed to attach and grow (**Figure 2A**).

Cells attached within a day and had epithelial characteristics. At low densities, they organized as colonies and proliferated in the plate to form a cellular monolayer. Cells were cuboidal and well-organized, as is commonly found in epithelial tissue (**Figure 2A**). In comparison, the cells eliminated during the purification process, *i.e.*, cells attached to the magnetic beads, had stromal characteristics specific to connective tissue. These cells had a spindle-shaped form and proliferated faster than epithelial cells. In two-dimensions, our primary cell culture protocol allows epithelial cells to retain their epithelial phenotype for up to six days (**Figure 2A**). However, with longer culture time, we observed a cell transition toward a “fibroblast-like” phenotype, as shown on day 8 in **Figure 2A**.

To confirm that purified cells in culture were epithelial cells, we performed western blots with specific antibodies targeting cytokeratin 8 and 18 (CK8/18), known to be specific to luminal epithelial cells, and vimentin, an intermediate filament found in non-muscle cells including fibroblast and endothelial cells (**Figure 2B**). After three days in culture, purified epithelial cells showed a high signal of CK8/18, indicating a high content of epithelial cells, and barely detectable levels of vimentin. Instead, vimentin was high in the cell fraction eliminated through differential plating (DP), a necessary step to avoid fibroblast contamination. CK8/18 remained high at six days, but vimentin levels increased over time in the purified epithelial cell fraction. This is consistent with results obtained by immunofluorescence analyses. Notably, 80% of the cells were positive for CK8/18 at three days, a ratio maintained up to six days in culture (**Figures 2C** and quantified in **D**). Cells positive for vimentin represented less than 10% of the cells after three days in culture, and slightly increased after six days. Altogether, these results indicate that the mammary epithelial cell purification protocol we performed leads to a significant enrichment of the epithelial cell compartment. However, the epithelial phenotype is lost over time after prolonged two-dimensional cell culture.

### Organoid Culture Recapitulates Mammary Gland Organization/Structure

**Figure 3:**
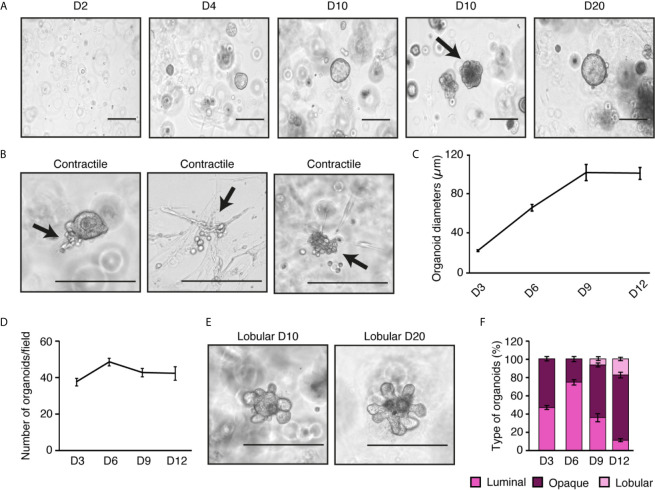
*Ex vivo* mammary epithelial cell organoids recapitulate the *in vivo* architecture of the mammary gland. **(A)** Brightfield visualization of organoids over 20 days in three-dimensional culture. At 10 days, spheroid-like organoids can be observed along with the beginning of lobular structures (black arrow). **(B)** Contractile structures are indicated by black arrows (see videos in **Supplemental Figure S1** to observe contractions). **(C)** Diameter measurements of organoids over time (45 organoids measured/day). **(D)** Number of visible organoids through time in three-dimensional culture. **(E)** Brightfield visualization of lobular organoids after 10 and 20 days in three-dimensional culture. **(F)** Percentage of luminal, opaque, and lobular organoids over time. For **(C, D, F)**, results are shown as mean ± SEM of one out of three independent experiments. Scale bars = 300 μm for **(A, B, E)**.

For long-term culture of primary mammary epithelial cells, we then tested our purified cells in three-dimensional cell culture to obtain primary mammary organoids. Briefly, cells purified during step 6 in **Figure 1A** were plated after differential plating in a Matrigel disk to allow cells to grow in three-dimensions. Organoids started to be easily visible after three days in culture and continued growing for several days (**Figures 3A–C**). Once visible, their numbers remained stable over time (**Figure 3D**), and they could be maintained in culture more than one month (data not shown).

These organoids differentiated into two major types. Luminal organoids have bigger lumens and are more spherical and brighter in brightfield visualization, as previously described by others (27). Opaque organoids are considered to have a smaller lumen and thus appear denser and more heterogeneous in shape. Recent studies have shown that organoids with lumen are mainly made of luminal epithelial cells whereas opaque organoids are primarily constituted of basal cells (27). Other structures associated with mammary gland biology started to be visible after a few days in culture. For instance, after six days, contractile cells were found (**Figure 3B**). They could organize themselves in the Matrigel disk (**Figure 3B**, panels 2 and 3) or be attached to an organoid (**Figure 3B**, panel 1). Their contraction started without any stimulation and the movement initiated by one cell seemed to enhance the contractions of nearby cells (see videos in **Supplemental Figure S1**). In the mammary gland, basal epithelial cells could be myoepithelial contractile cells to help with milk expulsion (16, 28). After 10 days of culture, lobular structures appeared (**Figure 3E**). These structures developed from the center of the organoids and expanded over time. Similar structures were found during the branching morphogenesis occurring at puberty and pregnancy (26).

We could also monitor how the different types of organoids changed in culture over time (**Figure 3F**). On day 3, an equivalent number of luminal and opaque organoids could be seen. While luminal organoids tended to be more frequent on day 6. On day 9, when lobular structure appears, the number of luminal organoids decreased, suggesting that luminal organoids are the ones in which lobular structures arise. Opaque organoids were stable between days 9 and 12, showing that they were not affected by the development of lobular structures. These results demonstrate that the current protocol to purify mammary epithelial cells for primary culture recapitulates the structures commonly found in the mammary gland.

### Cultured Epithelial Cells in Two- and Three-Dimensions Retain an Active Estrogen Signaling Pathway

**Figure 4:**
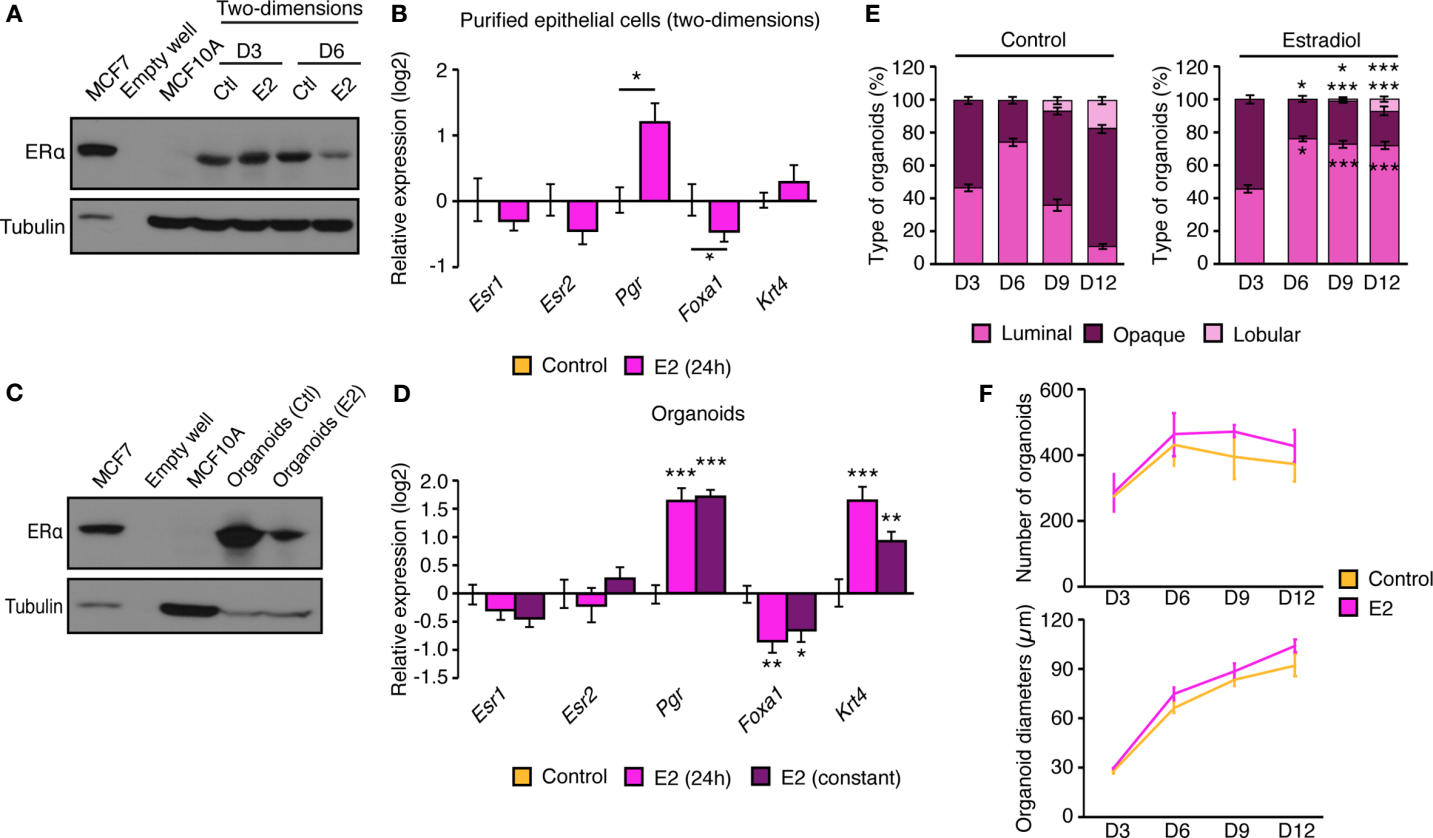
Primary mammary epithelial cells in two- and three-dimensions express ERα. **(A)** Western blot analysis of ERα protein expression in mammary epithelial cells in two-dimensional culture after 3 and 6 days in culture with E_2_ or vehicle. MCF7 human breast cancer cells and MCF10A human immortalized mammary epithelial cells were used as positive and negative controls for ERα protein expression, respectively. **(B)** qRT-PCR analysis of genes regulated by estrogens in two-dimensional culture following a 24 h treatment with E_2_ or vehicle. **(C)** Western blot analysis of ERα protein expression in mammary organoids after 12 days in culture. MCF7 human breast cancer cells and MCF10A human immortalized mammary epithelial cells were used as positive and negative controls for ERα protein expression, respectively. **(D)** qRT-PCR analysis of genes regulated by estrogens in primary mammary gland organoids following 24 h or 10 d of treatment with E_2_ or vehicle. For **(B, D)**, results are shown as mean ± SEM of three independent experiments performed at least in duplicate. **(E)** Percentage of luminal, opaque, and lobular organoids over time, maintained in culture with and without E_2_. Results are shown as the mean ± SEM of one out of three independent experiments. *p < 0.05; **p < 0.01; ***p < 0.001. **(F)** Number of visible organoids treated with E_2_ or vehicle. Results are shown as the mean ± SEM of two independent experiments performed in triplicate.

Because ERs expression is often lost in immortalized MECs (29), we wanted to validate that the current procedure for primary culture allowed the stable expression of these sex-steroid hormone receptors. We first assessed ERα protein expression levels in primary MECs cultured in two-dimensions (**Figure 4A**). We used MCF7, a human breat cancer cell line that exepresses ERα as positive control; for the negative control, we used MCF10A, a human non-tumorigenic mammary epithelial cell line that has no detectable expression of the receptor. After three and six days in two-dimensional culture, MECs conserved detectable ERα protein expression. Next, we wanted to make sure that the ERα signaling pathway was functional given its established role in the mammary gland (2). In two-dimensional culture, E_2_ treatment for 24 h did not significantly modulate *Esr1* or *Esr2* (**Figure 4B**); this is expected, since ERs have not been reported to modulate themselves at the transcriptional level in the mammary gland (30). However, E_2_ significantly induced the expression of the progesterone receptor (*Pgr*), a well-known ERα target gene (**Figure 4B**). In parallel, E_2_ treatment significantly repressed the expression of *Foxa1*, as ERα does *in vivo* (30). *Krt4*, reported to be induced by E_2_ *in vivo* in the mammary gland, was not regulated by this hormone in the current settings. The protein expression of ERα was also investigated in organoids after 12 days in culture. As for MECs, ERα protein was still expressed at significant levels (**Figure 4C**). The estrogenic response of mammary gland organoids was also tested, both with a transient E_2_ treatment of 24 h and following chronic activation of ERα (10 days; **Figure 4D**). In both contexts, *Pgr*, *Foxa1*, and *Krt4* were significantly modulated by E_2_ treatment as expected, with similar responses between treatments of 24 h or 10 days, indicating that the hormonal response remains functional in primary mammary organoids over time. Finally, we assessed the functional impact of E_2_ on the different types of organoids observed in three-dimensional culture over time. As shown in **Figure 3**, we observed similar levels of luminal and opaque organoids after three days in culture, while there was a peak in luminal organoids at day 6 in both the control and E_2_ treatment (**Figure 4E**). Interestingly, a shift was observed in the control condition, with a decrease in luminal organoids and the apparition of lobular organoids on days 9 and 12. E_2_ significantly impaired this process, favoring the maintenance of luminal organoids as the major type of organoids observed through time and decreasing the formation of lobular organoids. These changes in organoid structures are unlikely related to total organoid number nor diameter alterations, since no significant differences over time was observed with E_2_ treatment (**Figure 4F**). These results indicate that primary MECs obtained with our methods express ERα with a functional estrogen signaling pathway, and that E_2_ exposure significantly alters organoid formation and structural organization in three-dimensional culture.

### Optimization of Culture Conditions for Primary Epithelial Cell Metabolic Analyses

**Figure 5:**
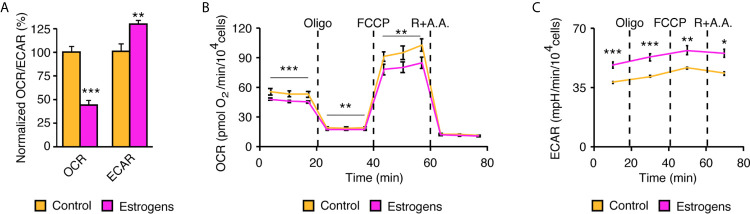
Reprogramming of mammary epithelial cell metabolism in two-dimensions by estrogens. **(A)** Extracellular flux analysis of mammary epithelial cells using an XFe96 Seahorse apparatus. Oxygen consumption rate (OCR), an indicator of mitochondrial respiration, and the extracellular acidification rate (ECAR), an indicator of aerobic glycolysis, are shown normalized to their respective controls. (n= 3 biologically independent samples). Metabolic flexibility of OCR **(B)** and ECAR **(C)** after injections of mitochondrial modulators. Dashed lines indicate when in the assay the different drugs were injected and followed by the metabolic response. Oligo, oligomycin; R, rotenone; AA, antimycin. **(A)** Results are shown as the mean ± SEM of one out of four independent experiments, each with 4-5 biological replicates per group. All experiments were performed in primary mammary epithelial cells in two-dimensions after 3 days in culture and 48 h of treatment with estradiol or vehicle. *p < 0.05; **p < 0.01; ***p < 0.001.

Given that lactation requires high energy levels, we hypothesized that sex-steroid hormones would reprogram mammary gland epithelial cell metabolism to support this process and that MECs in primary culture could be a good model to study this phenomenon. To examine the functional impact of estrogens on cell metabolism, we used an XFe96 extracellular flux analyzer to measure oxygen consumption rates (OCR) and extracellular acidification rates (ECAR). OCR allows the measurement of mitochondrial respiration. ECAR is a readout of lactate production and secretion, which are indicative of aerobic glycolysis, a metabolic pathway often hyperactivated in highly proliferative cells such as cancer cells (also known as the Warburg effect) (4). OCR and ECAR were assessed in mammary epithelial cells purified using the complete protocol we have described (**Figure 1A**). After 24 h in culture, fresh media without serum was added to the cells. After 48 h, to allow steroid deprivation, cells were then treated with vehicle or E_2_ for an additional 48 h before metabolic flux analysis. Interestingly, E_2_ treatment induces a significant change in the usage of these two pathways, which represent the two major cellular pathways for ATP synthesis. Indeed, the reliance of mammary epithelial cells on OCR decreased to favor aerobic glycolysis, as monitored by ECAR (**Figure 5A**).

The XFe96 apparatus has four injection ports that allow modulation of the mitochondrial machinery in real-time. In mammary epithelial cells, oligomycin first inhibits ATP synthase and blocks mitochondrial respiration coupled to ATP synthesis (**Figure 5B**). FCCP is then injected to allow maximal respiration of these cells, and finally rotenone and antimycin A allow a complete blockade of mitochondrial respiration, leaving only the non-mitochondrial respiration signal. Interestingly, E_2_ treatment decreased basal respiration (before injections), but also decreased the maximal respiration (reserve capacity) of mammary epithelial cells. In parallel, ECAR can be measured to study how cells reprogram their metabolism following inhibition of mitochondrial respiration (**Figure 5C**). Basal ECAR levels (before injections) were higher in E_2_-treated cells, and they remained at higher levels even after the different mitochondrial stresses. Overall, our results demonstrate that E_2_ reprograms mammary epithelial cell metabolism by promoting the Warburg effect.

### MS-Based Metabolomics in Primary Mammary Organoids Reveal a Specific Reprogramming of Metabolism by Estrogens

**Figure 6:**
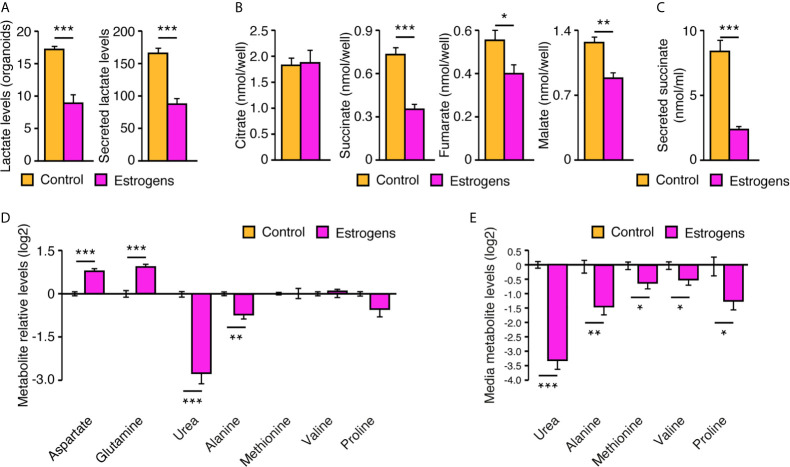
Reprogramming of cell metabolism by estrogens in mouse mammary organoids. After 12 days in culture, with media changed every 72h, organoids and extracellular media were harvested for GC-MS targeted metabolomics to measure lactate **(A)**, TCA cycle intermediates **(B)**, secreted succinate **(C)**, and other metabolite levels in organoids **(D)** or in the extracellular media **(E)**. Organoids were either treated with E_2_ or vehicle. All results are shown as the mean ± SEM of two independent experiments performed in triplicate. *p < 0.1; **p < 0.05; ***p < 0.001.

Organoids have been shown to be more complex structures and to alter cell metabolism compared to traditional monolayer cell culture (31–33). Thus, we hypothesized that our mouse mammary gland organoids could also have a different cell metabolism and a distinct metabolic response to estrogens compared to purified MECs in two-dimensions. To test this hypothesis, organoids were firstly treated with E_2_ or vehicle, then both organoids and the extracellular media were harvested for targeted metabolomics using gas chromatography - mass spectrometry (GC-MS). As XFe96 results in two-dimensions showed a Warburg effect with estradiol (**Figure 5**), we expected to observe an increase in lactate levels. Surprisingly, lactate levels in both organoids and media were significantly lower following E_2_ treatment (**Figure 6A**). To test the impact of E_2_ on the mitochondrial respiration in organoids, several tricarboxylic acid (TCA) cycle intermediates were also measured. We did not detect any changes in citrate levels, the first intermediate of the cycle, but downstream metabolites were all significantly decreased (**Figure 6B**). Intriguingly, one of these metabolites, succinate, was detected at high concentrations in the extracellular media (~10 nmol/ml) and was decreased by 3.6-fold following E_2_ treatment (**Figure 6C**). Metabolites from other pathways in organoids were also quantified, notably showing a significant decrease in urea and alanine levels following E_2_ treatment (**Figure 6D**). This decrease in organoids was paralleled by a significant decrease of urea and alanine levels in the extracellular media by 9- and 2.6-fold, respectively (**Figure 6E**). On the contrary, other metabolite levels in organoids were increased following E_2_ treatment, like aspartate and glutamine (**Figure 6D**). Altogether, these results demonstrate that ERα activation reprograms cell metabolism. Important differences were also observed compared to two-dimensional culture, suggesting that how ERα reprograms metabolism is dependent on the three-dimensional cellular organization and cell-cell interactions.

## Discussion

In this study, we describe in detail a FACS-free protocol to purify and culture epithelial cells from the mouse mammary gland. This protocol can be used to obtain cells for primary culture in both two- and three-dimensions, in which they can form complex organoid structures that recapitulate the mammary gland structure *in vivo*. Furthermore, we demonstrate that these MECs retain ERα expression, allowing *ex vivo* studies of this critical hormonal signaling pathway in the mammary gland. Finally, as a proof-of-principle, we show that these cells can be used for metabolic studies in primary culture and that ERα activation promotes specific metabolic reprogramming of primary mammary epithelial cells in two- and three-dimensions. To our knowledge, this is the first time ERα is shown to modulate cell metabolism in MECs and mammary organoids.

Mouse MECs are commonly purified using FACS, differential centrifugations, or antibody selection using magnetic beads. For simplicity and maximal purity, the protocol we describe focused on the latter type of protocol. Regarding the cell numbers obtained after purification, our protocol is comparable with FACS methods. We found about half a million MECs per mammary gland, which is similar to what was described in FVB mice by Smalley et al. (34). Our percentage of Ma-CFC and MRU populations is higher than what has been found using other protocols (3.88% MRU in total cells for us compared to an MRU frequency of 1 in 200 sorted cells using a sorting strategy) (34); this difference could be explained by the fact that we only used two markers (CD24 and CD49f). Furthermore, many parameters could influence these numbers, like the age of the mice (around 8 to 10 weeks whereas our average age was 20 weeks) or the strain (34). For example, some stromal cells in C57BL/6 mice, especially in young mice, express CD24, which decreases the ability to resolve the luminal and basal cell populations on a flow dot plot (34). Otherwise, the protocol we describe has several advantages, including no requirement of specialized equipment such as a flow cytometer, the high purity of MECs obtained for primary culture, and the maintained expression of ERα and estrogenic response through time.

ERα is essential for mammary gland development. In fact, its knockout (KO) blocked mammary gland development after puberty, with a lack of epithelial branching and lobuloalveolar development in *Esr1* KO mice (35). Following puberty, sex-steroid hormones promote ductal elongation in mouse mammary gland (36). E_2_ is known to stimulate growth and expansion of the ducts into the mammary fat pad as ERα^+^ cells promote proliferation of surrounding cells by a paracrine mechanism (2). We had assumed that E_2_ would increase branching morphogenesis in our organoid system, but we observed the opposite result. In our experiments, other hormones essential for the proper functioning of the mammary gland were missing. For instance, progesterone is known to increase the proliferation of MECs during the diestrus phase of the mouse’s cycle whereas our culture condition—with only a peak of estrogen—is more similar to the less-proliferative proestrus phase. Indeed, ovarian hormones in the absence of pituitary hormones have little or no mammogenic activity in rodents (2, 37). Consequently, future studies with more complex hormone combinations are required to fully recapitulate mammary gland organogenesis *ex vivo*.

Despite being a major metabolic investment for females, the metabolic reprogramming of MECs during lactation as well as the different regulatory factors required to sustain cell metabolism for the lactation process remain mostly unknown. Two key signaling pathways, namely the AMP-activated kinase (AMPK) and the mechanistic target of rapamycin (mTOR), have been linked to this metabolic regulation (5, 38). For example, AMPK activation by pharmacological compounds has been shown to decrease lipid synthesis through phosphorylation of acetyl-CoA carboxylase (ACC) and to decrease processing of the SREBP1 lipogenic transcription factor (37, 39). Not much is known about how estrogens and their receptors participate in this reprogramming of cell metabolism in the mammary gland. Recent research by the Maggi group has clearly established that ERα is a key determinant that promotes lipid synthesis using amino acids as a source of fuel in hepatocytes in females, which distinguish liver metabolism between males and females (40). In the liver, this leads to an energy partition strategy that is thought to be the result of selective pressure to tailor reproductive functions to the nutritional status (40). In prostate cancer, androgens—through activation of their receptor AR—have also been shown to be major orchestrators of specific metabolic pathways, such as mitochondrial respiration, lipid synthesis and usage, and glycolysis modulation (4, 20, 21, 41–43). Thus, sex-steroid hormones appear to be key modulators of cell metabolism and to have specific functions in distinct peripheral tissues.

We showed that the estrogen signaling pathway promotes aerobic glycolysis over mitochondrial respiration in primary MECs grown in two-dimensions, a phenomenon known as the Warburg effect. Surprisingly, we observed a different modulation of metabolism when MECs were grown in organoids, with a negative regulation of lactate production and secretion by E_2_ in three-dimensional culture. Contrary to MECs grown in two-dimensions, organoids are composed of several cell types, including epithelial luminal and basal cells, and thus represent a more complex environment. E_2_ does not alter the number of organoids and their diameters compared to the vehicle (**Figure 4F**), but it does alter the types of organoids (**Figure 4E**). Consequently, it most probably alters the relative fraction of the different cell types composing these organoids, as well as the cell-cell interactions occuring in these organoids. In addition, the three-dimensional structure probably promotes nutrients and oxygen-gradients that will also modulate cell metabolism, as this was shown to be the case in other cellular contexts (31). In organoid culture, E_2_ treatment also induced significant alterations of metabolite levels from several metabolic pathways, including the TCA cycle, urea cycle, and amino acid metabolism. Succinate, fumarate, and malate of the TCA were all significantly decreased by E_2_ treatment, but not citrate levels, possibly suggesting a global decrease in mitochondrial respiration. The important changes of urea intra-cellular and secreted levels also suggest an important modulation of the urea cycle in MECs. Given that the results shown herein are from a combination of different cell types interacting together, further mechanistic studies are required to fully understand how ERα reprograms cell metabolism, as it is highly probable that it reprograms both luminal and basal cell metabolism in a direct and indirect (paracrine) manner, respectively. In any case, it is clear that ERα is an important regulator of cellular metabolism in mammary organoids.

In conclusion, purified MECs can be used both for two- and three-dimensional *ex vivo* culture analyses, and they recapitulate different mammary gland structures when cultured to form organoids. These purified MECs are also compatible with sex-steroid hormone signaling studies and their impact on normal mammary epithelial cell metabolism. This study could provide a simple and evolutive tool to better understand the relation between hormones and metabolism in the mammary gland. Notably, our results demonstrate that the estrogen signaling pathway is a powerfull modulator of cell metabolism, but future studies are required to fully decipher the metabolic functions of ERα in mammary glands.

## Data Availability Statement

The raw data supporting the conclusions of this article will be made available by the authors, without undue reservation.

## Ethics Statement

The animal study was reviewed and approved by Université Laval Research and Ethic Animal Committee.

## Author Contributions

Conception and design of the experiments: AL and EA-W. Collection, assembly, analysis and interpretation of data: AL, CJ, CW, LB, DB, IL, MP, and EA-W. Drafting the article: AL and EA-W. Revising the manuscript for critically important intellectual content: AL, CJ, CW, LB, DB, IL, MP, and EA-W. Study supervision: EA-W. All authors contributed to the article and approved the submitted version.

## Funding

This work was supported by funding to MP and EA-W from the National Sciences and Engineering Research Council of Canada (NSERC; RGPIN-2015-05413 and RGPIN-2019-04740, respectively). The acquisition of the extracellular flux analyzer was supported by a John R. Evans Leaders equipment and infrastructure grant (#33805) from the Canada Foundation for Innovation (CFI) awarded to MP. AL received a scholarship from the Fondation du CHU de Québec - Université Laval. MP is a Junior 2 scholar from the Fonds de recherche du Québec-Santé (FRQS) and IL a Junior 1 Scholar. IL was also supported by a John R. Evans Leaders Fund from the CFI (#37996). EA-W holds a Tier 2 Canada Research Chair from the Canadian Institutes of Health Research on targeting metabolic vulnerabilities in hormonal-dependent cancers. EAW was also supported by a John R. Evans Leaders Fund from the CFI (#38622).

## Conflict of Interest

The authors declare that the research was conducted in the absence of any commercial or financial relationships that could be construed as a potential conflict of interest.

## Publisher’s Note

All claims expressed in this article are solely those of the authors and do not necessarily represent those of their affiliated organizations, or those of the publisher, the editors and the reviewers. Any product that may be evaluated in this article, or claim that may be made by its manufacturer, is not guaranteed or endorsed by the publisher.
