# Supplementary material for: A FACS-Free Purification Method to Study Estrogen Signaling, Organoid Formation, and Metabolic Reprogramming in Mammary Epithelial Cells
Source: Front Endocrinol (Lausanne). 2021 Aug 12;12:672466. doi: 10.3389/fendo.2021.672466 (PMC8397380; doi:10.3389/fendo.2021.672466)
Supplement: Supplementary file 1 [file DataSheet_1.docx]

***Supplementary Material***

**Inventory:**

**Supplementary Figure 1 (ppt)**

**Supplementary Table 1**

**Supplementary Figure 1. A.** Video of contractile structures attached to an organoid at a 40X magnification. **B.** Video of a self-organized contractile structure at a 10X magnification.

**Supplementary Table 1.** Mouse qRT-PCR primers

| Primer | Sequence | Organism |
| --- | --- | --- |
| *Tbp* Forward | GTTGGGCTTCCCAGCTAAGT | *Mus musculus* |
| *Tbp* Reverse | CACAAGGCCTTCCAGCCTTA | *Mus musculus* |
| *Pum1* Forward | AACATCGATGGCCTACAGGG | *Mus musculus* |
| *Pum1* Reverse | GACCAGGTCTTCTCTGCACC | *Mus musculus* |
| *Actn* Forward | GTTGGGTCCCGAAGAGTTCA | *Mus musculus* |
| *Actn* Reverse | CGGTTGGGGTCTACAATGCT | *Mus musculus* |
| *Esr1* Forward | CATAACAGCCTCGGAACGGA | *Mus musculus* |
| *Esr1* Reverse | CCTCTCCTGGCATCACAAGG | *Mus musculus* |
| *Esr2* Forward | AAGAAGCAGGGGAAGTAAGGC | *Mus musculus* |
| *Esr2* Reverse | CTTTCCTCTGCAGGCATTCCA | *Mus musculus* |
| *PgrB* Forward | GCTTGGACTCAGGTCCCTTC | *Mus musculus* |
| *PgrB* Reverse | GGGCTCTGGAATTTCTGCCT | *Mus musculus* |
| *Foxa1* Forward | CAGCTGCATCTGAAAGGGGA | *Mus musculus* |
| *Foxa1* Reverse | GCACGGGTCTGGAATACACA | *Mus musculus* |
| *Krt4* Forward | CTTCAACGGTCGAGGAGGTC | *Mus musculus* |
| *Krt4* Reverse | GTCAGCAGGCTCTGGTTGAT | *Mus musculus* |
