# Supplementary figures and images for: A FACS-Free Purification Method to Study Estrogen Signaling, Organoid Formation, and Metabolic Reprogramming in Mammary Epithelial Cells
Source: Front Endocrinol (Lausanne). 2021 Aug 12;12:672466. doi: 10.3389/fendo.2021.672466 (PMC8397380; doi:10.3389/fendo.2021.672466)

## Slide 1
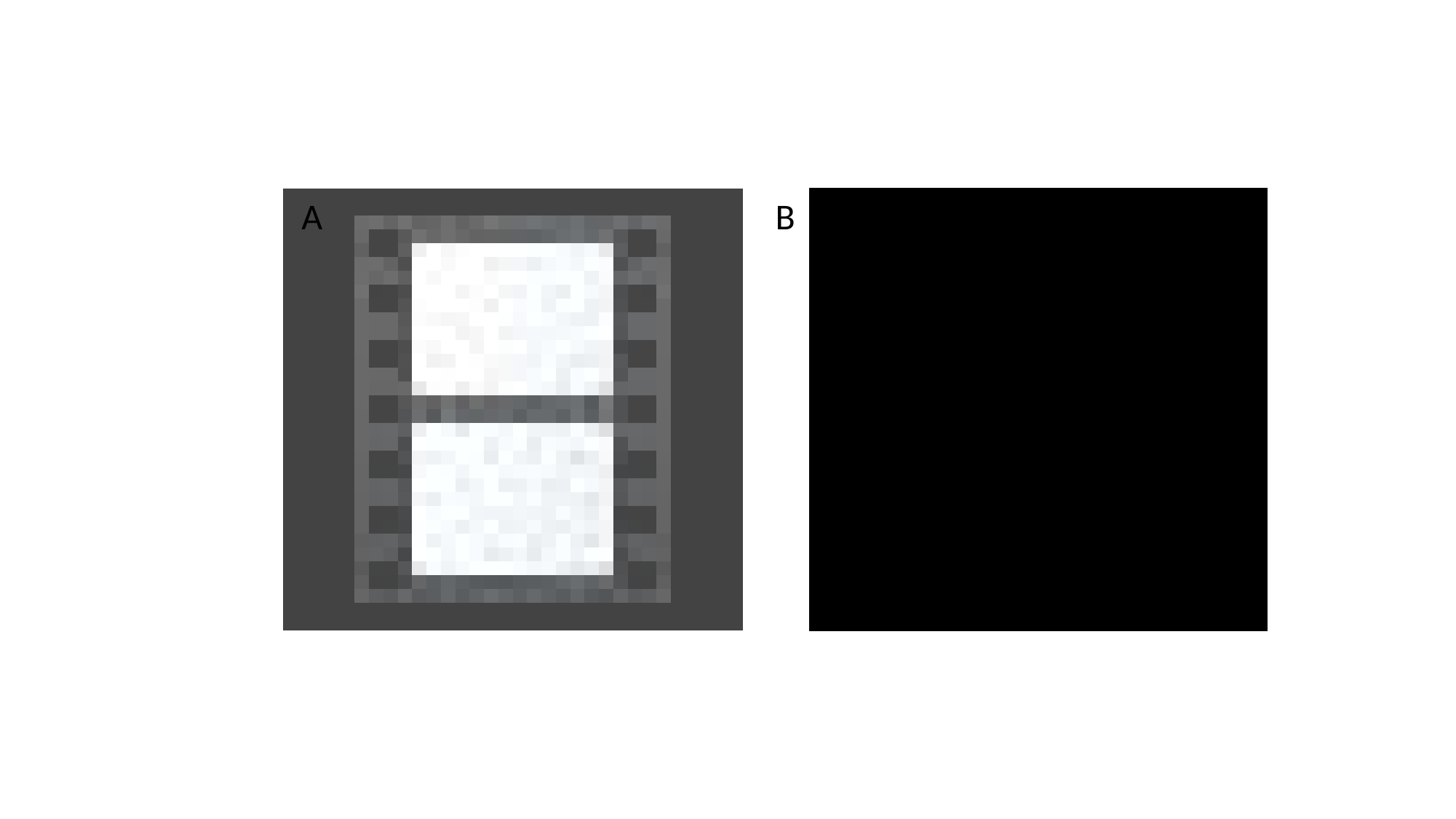

A
B

Supplement: Supplementary file 2 [file Presentation_1.pptx]
